# A case report of neonatal renal pseudohypoaldosteronism type I caused by a *de novo* variant in the *NR3C2* gene

**DOI:** 10.3389/fped.2026.1774632

**Published:** 2026-04-30

**Authors:** Hongcai Ma, Huijiao Han, Yanyan Liu, Yong An

**Affiliations:** Department of Laboratory Medicine, Xingtai Ninth Hospital, Xingtai City, China

**Keywords:** case report, genes, hyperbilirubinaemia, *NR3C2* protein, pseudohypoaldosteronism

## Abstract

Pseudohypoaldosteronism type I (PHA I) is a rare hereditary disorder of mineralocorticoid resistance, with the renal form predominantly caused by *NR3C2* variants. We report a male neonate presenting with severe hyperbilirubinaemia who was subsequently diagnosed with renal PHA I. Born at 38⁺⁶ weeks, he was admitted on day 7 for jaundice. Laboratory evaluation revealed refractory hyponatraemia, markedly elevated plasma aldosterone and renin levels, and ineffective conventional electrolyte therapy. Whole-exome sequencing identified a *de novo* heterozygous nonsense variant, c.1954C>T (p.Arg652*), in *NR3C2*, confirming the diagnosis. Aggressive sodium supplementation corrected the electrolyte imbalance, and follow-up showed normal growth and stable serum sodium. This case underscores the need to consider inherited renal tubulopathies in neonates with refractory hyponatraemia and highlights the pivotal role of genetic testing in precise diagnosis.

## Introduction

Pseudohypoaldosteronism type I (PHA I) is a rare disorder of mineralocorticoid resistance characterised by renal salt wasting, hyponatraemia, hyperkalaemia, and metabolic acidosis, together with markedly elevated plasma renin activity and aldosterone levels. Its estimated incidence is approximately 1 in 80,000 live births. Renal PHA I accounts for approximately 50% of cases and is most commonly caused by heterozygous loss-of-function variants in the *NR3C2* gene, which encodes the mineralocorticoid receptor, and follows an autosomal dominant inheritance pattern (1). Variants in *NR3C2* result in mineralocorticoid receptor dysfunction, rendering the distal renal tubules unresponsive to aldosterone, thereby impairing sodium reabsorption, inducing hyponatraemia, and triggering compensatory activation of the renin–angiotensin–aldosterone system (RAAS) (2). The disease often presents in the neonatal period or early infancy, and its clinical manifestations may be easily misinterpreted as neonatal infection or gastrointestinal disease, leading to delayed diagnosis. We describe a case of PHA I in which severe jaundice was the initial clinical presentation, highlighting the need for increased awareness of this condition among clinicians.

## Case report

### Ethics approval and informed consent

This study was approved by the Ethics Committee of Xingtai Ninth Hospital and conducted in accordance with the Declaration of Helsinki. The patient's parents (guardians) provided written informed consent for the use of clinical data and genetic testing results for research and publication purposes.

### Basic information

The patient was a 7-day-old male neonate admitted for progressively worsening jaundice over 7 days. He was the second child of a second pregnancy (G2P2), with a birth weight of 3.41 kg. Apgar scores were 10 at both 1 and 5 min, and the perinatal history was unremarkable. Jaundice developed on day 7 after birth, initially involving the face and subsequently spreading to the trunk, with progressive intensification. No fever or convulsions were observed. Transcutaneous bilirubin reached a peak level of 21.0 mg/dL. On admission, he was diagnosed with neonatal hyperbilirubinaemia, neonatal infectious jaundice, and neonatal omphalitis.

### Physical examination on admission

Vital signs, including body temperature, respiratory rate, and heart rate, were stable. The skin and mucous membranes appeared pale yellow with mottling, and the feet were mildly cyanotic. A small amount of purulent discharge was observed in the umbilical fossa. Cardiac, pulmonary, and abdominal examinations revealed no obvious abnormalities. Limb muscle tone was mildly decreased. Urine output was 4.75 mL/kg/h.

### Auxiliary examinations

Severe indirect hyperbilirubinaemia was observed, with a total bilirubin level of 342.1 μmol/L and an indirect bilirubin level of 331.82 μmol/L. Serum sodium levels persistently ranged from 127.1 to 131.1 mmol/L (reference range: 135–145 mmol/L), whereas serum potassium remained within the normal range at 4.8 mmol/L (reference range: 3.5–5.1 mmol/L). Hormonal evaluation demonstrated marked activation of the RAAS, with aldosterone 3,178.6 pg/mL (reference range: 10–160 pg/mL), renin 466.29 pg/mL (reference range: 4–24 pg/mL), angiotensin II 1,789.04 pg/mL (reference range: 25–129 pg/mL), and plasma renin activity 6.7 ng/mL/h (reference range: 0.15–2.33 ng/mL/h). Urine electrolyte analysis showed elevated urinary sodium at 98 mmol/L and urinary potassium at 25 mmol/L, indicating renal sodium wasting. Arterial blood gas analysis indicated metabolic acidosis, with a pH of 7.30, bicarbonate level of 16 mmol/L, and extracellular base excess of −6.0 mmol/L. Ultrasound examinations of the cranium, heart, gastrointestinal tract, and urinary system revealed no structural abnormalities that could account for the hyponatraemia.

### Treatment course and diagnostic dilemma

Upon admission, the patient was immediately treated with blue light phototherapy (8–12 h/day for a total of 5 days), antibiotics (ceftriaxone 50 mg/kg/day by intravenous infusion for 7 days), and intravenous fluid support. Due to refractory hyponatraemia, continuous infusion of 3% sodium chloride via a micro-pump was initiated at 2–4 mmol/kg/day; however, serum sodium levels could not be corrected to the normal range. Serial monitoring showed that despite aggressive sodium supplementation, serum sodium fluctuated between 127 and 132 mmol/L, potassium levels remained within the normal range, and both aldosterone (>3,000 pg/mL) and renin (>400 pg/mL) were markedly elevated (Table 1). This persistent hyponatraemia resistant to sodium replacement, together with extreme activation of the RAAS, strongly suggested an underlying renal salt-wasting disorder.

**Table 1:** Changes in electrolytes and RAAS parameters during hospitalization.

Time	Sodium (mmol/L)	Potassium (mmol/L)	Aldosterone (pg/mL)	Renin (pg/mL)	Angiotensin II (pg/mL)
Day 1	127.1	4.8	3,178.6	466.29	1,789.04
Day 3	129.5	4.5	2,980.2	450.15	1,650.33
Day 7	131.1	4.3	3,105.8	442.70	1,702.91

The reference ranges for the laboratory indicators are as follows: serum sodium, 135–145 mmol/L; serum potassium, 3.5–5.1 mmol/L; aldosterone, 10–160 pg/mL; renin, 4–24 pg/mL; angiotensin II, 25–129 pg/mL.

### Diagnosis and differential diagnosis

**Figure 1:**
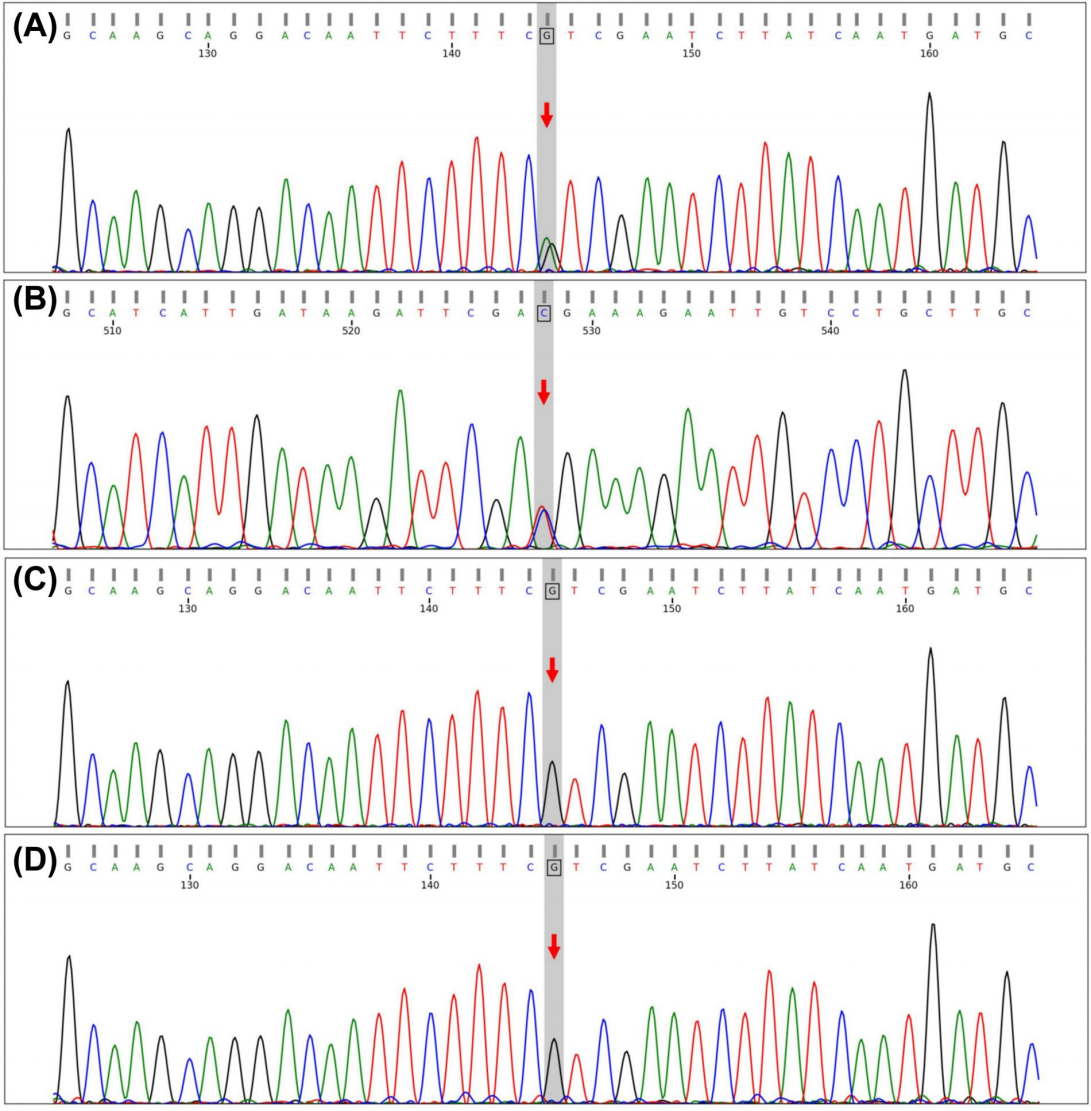
Sanger sequencing confirmation of the *NR3C2* c.1954C>T (p.Arg652) variant in the family pedigree. Panels **(A,B)** show the proband's forward and reverse sequencing results, respectively, while Panels **(C,D)** show the sequencing results of the father and mother, respectively.

(1) Preliminary Diagnosis: Based on the initial clinical presentation and laboratory findings, the patient was preliminarily diagnosed with neonatal hyperbilirubinaemia, neonatal omphalitis, and neonatal hyponatraemia of unclear aetiology. (2) Differential Diagnosis: Given the persistent hyponatraemia, salt-wasting disorders were considered, including adrenal insufficiency, salt-wasting congenital adrenal hyperplasia (CAH), and Bartter syndrome. Although cortisol levels were slightly reduced, androgen levels and 17-hydroxyprogesterone were within normal ranges, making CAH unlikely. Moreover, the absence of hyperkalaemia suggested functional impairment at the level of the mineralocorticoid receptor rather than primary aldosterone deficiency. (3) Final Diagnosis: Whole-exome sequencing (WES) identified a heterozygous nonsense variant in the *NR3C2* gene, NM_000901.5:c.1954C>T (p.Arg652*) (Figure 1). This pathogenic variant introduces a premature stop codon, leading to truncation of the mineralocorticoid receptor within the ligand-binding domain and subsequent loss of function. Parental testing confirmed that both parents were wild-type at this locus, indicating a *de novo* variant. A review of the literature and public databases indicated that this variant has been previously reported in the Human Gene Mutation Database (HGMD) and in ClinVar, where it is classified as pathogenic (3). Based on the clinical presentation and genetic findings, a definitive diagnosis of PHA I was established.

### Treatment and outcome

**Figure 2:**
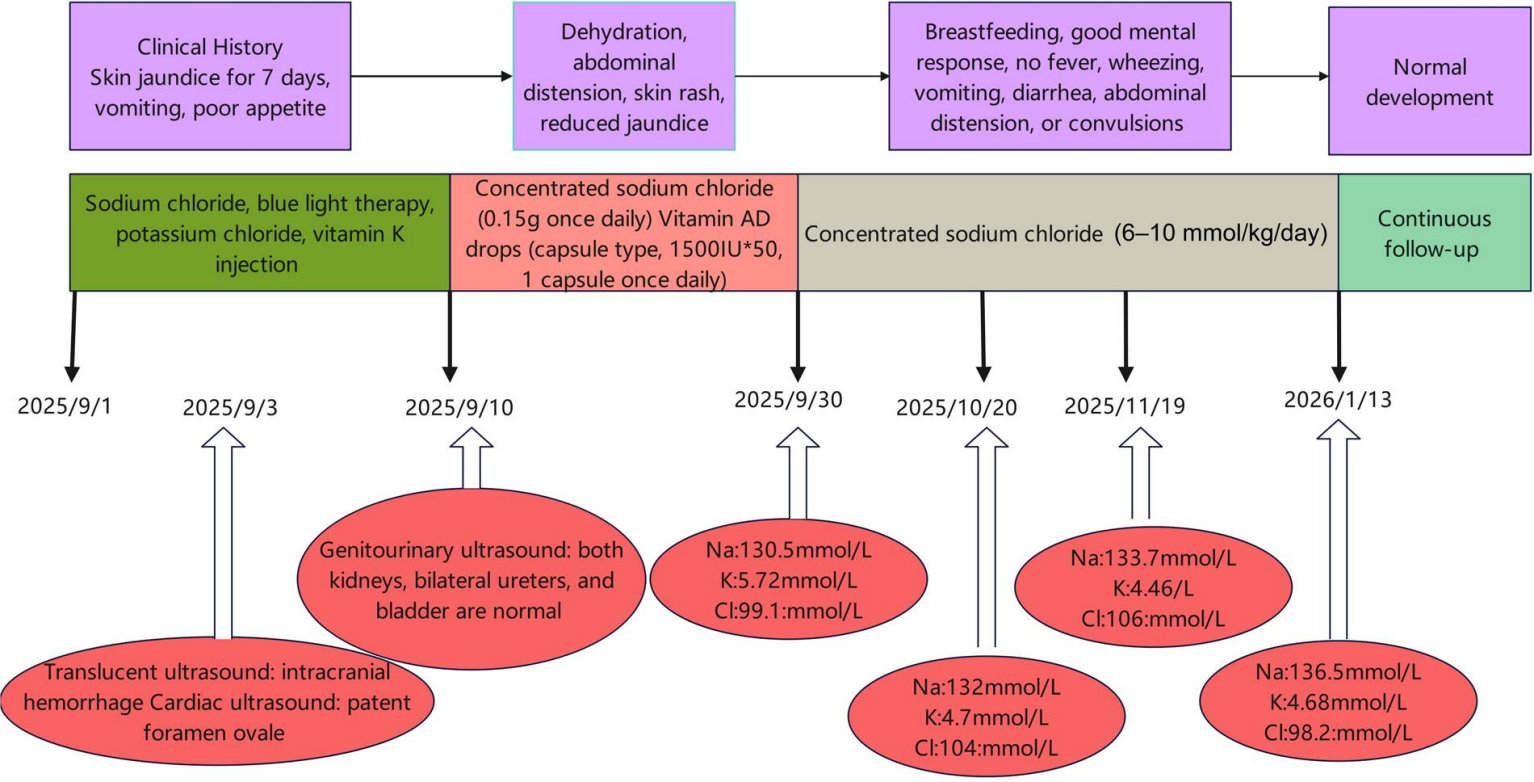
Timeline of the treatment process.

Before a definitive diagnosis, the patient received empirical sodium supplementation, including intravenous sodium chloride at approximately 2–4 mmol/kg/day (specifically, 3.4 mmol/kg/day); however, hyponatraemia persisted. Following genetic confirmation of PHA I, sodium supplementation was increased to an adequate weight-based dose (6–10 mmol/kg/day; specifically, 8.4 mmol/kg/day), resulting in gradual correction of electrolyte abnormalities. During follow-up, serum electrolytes and growth were monitored every 1–3 months. At 6 months of age, growth was appropriate, serum sodium levels were stable at 135–138 mmol/L, and RAAS-related markers had declined, although they remained above the normal range (Figure 2).

## Discussion

Renal PHA Ⅰ is a rare disorder caused by loss-of-function variants in the *NR3C2* gene, which encodes the mineralocorticoid receptor and is located on chromosome 4q31.1–31.2. In this study, a heterozygous nonsense variant, c.1954C>T (p.Arg652*), introduces a premature stop codon, resulting in truncation of the receptor within the ligand-binding domain and consequent loss of function. This defect leads to renal tubular resistance to aldosterone, excessive sodium wasting, and compensatory activation of the RAAS (3, 4). We describe a neonatal case presenting initially with hyperbilirubinaemia and subsequently diagnosed as PHA I through genetic testing. These molecular and clinical findings broaden the recognised phenotypic spectrum and underscore the value of precision genetic diagnosis.

The *NR3C2* gene encodes the mineralocorticoid receptor, a key mediator of aldosterone signalling. To date, more than 200 pathogenic *NR3C2* variants have been reported in the HGMD and ClinVar, with nonsense variants accounting for approximately 15% (5). The c.1954C>T (p.Arg652*) variant identified in this patient introduces a premature stop codon within the DNA-binding domain (DBD) of the mineralocorticoid receptor, resulting in truncated protein expression and loss of transcriptional regulatory activity. Similar to previously reported DBD missense variants, such as c.2441T>G (p.Leu814Trp) (5), and splice-site variants, including IVS-2 c.1757+1G>C (6), this disruption leads to renal tubular resistance to aldosterone, impaired sodium reabsorption, and compensatory activation of the RAAS (5). Clinically, PHA I typically presents in the neonatal period with salt wasting, hyponatraemia, hyperkalaemia, and metabolic acidosis. In contrast, this patient showed refractory hyponatraemia with marked RAAS activation but without substantial hyperkalaemia, consistent with previous reports (5). Notably, severe indirect hyperbilirubinaemia was the initial manifestation, with hyponatraemia detected incidentally, potentially delaying diagnosis. These findings suggest that haploinsufficiency caused by nonsense variants may produce phenotypes distinct from missense variants, possibly influenced by residual receptor function, genetic background, or epigenetic factors. Similar presentations have also been described (7), highlighting the phenotypic heterogeneity of *NR3C2*-related PHA I.

Hyperbilirubinaemia in this patient may represent a coincidental finding or a possible multisystem manifestation of PHA I rather than a confirmed causal effect. Although severe hyponatraemia might transiently influence hepatic enzyme activity or bile excretion, this association remains speculative and insufficiently supported (8). Compared with cases reported by Zhu Hongdan et al. (5), this case emphasises that PHA I should be considered in neonates with refractory hyponatraemia, regardless of potassium levels, especially when RAAS activation is evident. When clinical manifestations are non-specific and the response to conventional treatment is suboptimal, genetic testing plays a decisive role in the diagnosis of rare monogenic diseases (9). In this study, WES identified a pathogenic variant, which was further confirmed as *de novo* by parental verification. A review of HGMD, ClinVar, and PubMed indicated that the NR3C2 c.1954C>T (p.Arg652*) variant has been previously reported and classified as pathogenic. From a genetic counselling perspective, the *de novo* origin indicates a very low recurrence risk for siblings, whereas the patient's offspring carry a 50% risk of inheritance due to autosomal dominant transmission (3, 9).

The cornerstone of PHA I management is adequate sodium supplementation. During the neonatal period, prompt intravenous or oral sodium replacement is required to correct hyponatraemia and support normal growth and development (5). In this case, initial empirical supplementation prior to diagnosis was insufficient (approximately 3.4 mmol/kg/day) and failed to correct hyponatraemia. Following genetic confirmation, sodium supplementation was increased to an adequate weight-based dose (approximately 8.4 mmol/kg/day), resulting in gradual normalization of electrolyte levels. During follow-up to 6 months of age, serum sodium levels remained stable within the normal range, and growth was appropriate. Notably, some patients with PHA I, particularly the renal form, may develop partial renal tubular compensation with age, leading to clinical improvement and, in rare cases, discontinuation of sodium supplementation after childhood (5). Nevertheless, early and adequate sodium replacement is essential to prevent hyponatraemia-related neurological injury, and individualised long-term follow-up remains crucial.

Compared with previously reported cases of PHA I, this case demonstrates notable clinical value in several respects. First, it highlights an atypical presentation characterised by severe indirect hyperbilirubinaemia as the initial manifestation and refractory hyponatraemia without significant hyperkalaemia, thereby broadening the recognised phenotypic spectrum of the disorder. Second, it illustrates a precision diagnostic pathway in which WES enabled timely and accurate diagnosis in a clinically challenging setting, helping to avoid ineffective treatments and guide long-term management and genetic counselling. Genetic analysis further confirmed a *de novo* variant, indicating a low recurrence risk for siblings, while the patient's future offspring carry a 50% risk of inheritance (10–12). Importantly, this case underscores that PHA I should be considered in neonates with refractory hyponatraemia regardless of potassium levels, and that early assessment of RAAS indicators combined with genetic testing is essential for accurate diagnosis and optimal management (13).

## Conclusion

Renal PHA I is a rare and potentially life-threatening disorder. Clinicians should maintain a high index of suspicion for neonates presenting with refractory hyponatraemia and marked RAAS activation. Early recognition and confirmation through genetic testing are essential for guiding appropriate long-term management and improving outcomes. This case highlights the pivotal role of precision medicine in the diagnosis and treatment of complex neonatal disorders.

## Data Availability

The original contributions presented in the study are included in the article/Supplementary Material, further inquiries can be directed to the corresponding author/s.
